# Wisdom psychotherapy in adjustment disorders. Results of a randomized controlled study

**DOI:** 10.1192/j.eurpsy.2025.2147

**Published:** 2025-08-26

**Authors:** M. Linden

**Affiliations:** Department of Psychosomatic Medicine, Charité University Medicine Berlin, BNerlin, Germany

## Abstract

**Introduction:**

Wisdom is a capacity which is needed to cope with difficult situations in life. Wisdom can be trained like other capacities. Wisdom psychotherapy has been developed as a method of cognitive behavior therapy to help patients who are stuck in negative life experiences, or confronted with unsolvabale dilemmas.

**Objectives:**

Test the efficacy of wisdom psychotherapy in patients with adjustment disorders.

**Methods:**

patients with adjustment disorders (>18 on the ADNM8 scale) were randomly assigned to group wisdom therapy (WT: N=114), or group behavioral activation therapy (BA: N=109). Additionally a matched group of patients was build, which were not included in any study procedures but underwent routine treatment only (RT: N=114). Wisdom was measured with the Multidimensional Wisdom Competency Scale (MWC15).

**Results:**

There was an increase on the MWC15 of 5.3 in the wisdom group as compared to 0.4 in the activation group and 0,2 in the routine group. This is statically sigificant in the pre-post comparison and in the time/goup interaction (F4,42, p=0,13). The ADNM8 score, the SCL90 GSI, the BDI score decreased, with a trend for more side effects in the wisdom than the activity group.

**Conclusions:**

The results confirm that wisdom psychotherapy can make a difference in the improvement of wisdom capacities. Wisdom therapy, different from other psychotherapies, does not aim at increasing hedonic or symptom free wellbeing but rather eudaimonic wellbeing in order to teach patients to live a decent and successful life.

**Disclosure of Interest:**

None Declared

